# Polyphenols and Human Health: The Role of Bioavailability

**DOI:** 10.3390/nu13010273

**Published:** 2021-01-19

**Authors:** Chiara Di Lorenzo, Francesca Colombo, Simone Biella, Creina Stockley, Patrizia Restani

**Affiliations:** 1 Department of Pharmacological and Biomolecular Sciences, Università degli Studi di Milano, 20133 Milan, Italy; francesca.colombo1@unimi.it (F.C.); simone.biella@unimi.it (S.B.); 2 The Australian Wine Research Institute (AWRI), Glen Osmond 5064, Australia; creina.stockley@awri.com.au

**Keywords:** polyphenols, bioavailability, beneficial effects, human studies

## Abstract

Polyphenols are a group of phytochemicals with potential health-promoting effects. They are classified as flavonoid (flavonols, flavanols, flavones, flavanones, isoflavones, and anthocyanins) and non-flavonoid molecules (phenolic acids, hydroxycinnamic acids, lignans, stilbenes, and tannins). Although an increasing number of trials have shown a correlation among polyphenol consumption and a reduction in risk factors for chronic diseases, discrepancies in explaining their positive effects have been found in terms of the bioavailability. In fact, polyphenols show a low bioavailability due to several factors: interaction with the food matrix, the metabolic processes mediated by the liver (phase I and II metabolism), intestine and microbiota. On the other hand, the biological activities of phenol compounds may be mediated by their metabolites, which are produced in vivo, and recent studies have confirmed that these molecules may have antioxidant and anti-phlogistic properties. This review discusses the studies performed in vivo, which consider the polyphenol bioavailability and their different food sources. Factors influencing the biological effects of the main classes of polyphenols are also considered.

## 1. Introduction

**Figure 1:**
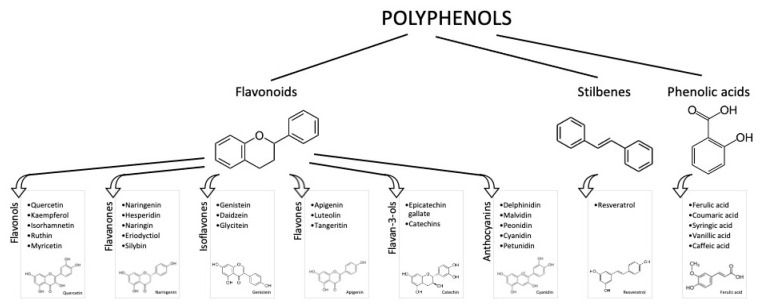
Polyphenol classes and chemical structures of some of their main compounds.

It is known that an appropriate diet and lifestyle are essential for preserving well-being and preventing illnesses. Due to their abundance in foods derived from plants (e.g., vegetables, fruits, and beverages) and their potential antioxidant activity, polyphenols have been considerably studied in the past years as adjuvants in attenuating the risk factors for disabling diseases (mainly cardiovascular disease (CVD), diabetes, cancer, and cognitive disorders) [1]. Polyphenols are phenylpropanoids synthetized by plants as secondary metabolites, in adverse situations, such as in the presence of pathogens or under adverse climatic conditions. More than 8000 phenolic molecules have been identified, which must contain at least one aromatic nucleus and one or more -OH groups [2]. Polyphenols are commonly categorized as flavonoids, characterized by a C6-C3-C6 structure, and non-flavonoids. Flavonoids generally present in foods are anthocyanins, flavonols, flavan-3-ols, flavones, isoflavones, flavanones, and stilbenes (Figure 1).

Most flavonoids found in foods are conjugated with sugars, acids, or alcohols. Non-flavonoids include phenolic acids, in particular, hydroxybenzoic acids (i.e., vanillic and gallic acids), and cinnamic acids (i.e., ferulic and caffeic acids). All of these molecules have proven to have biological activities [3,4]; however, most of them have been shown using in vitro models and pure compounds, where the metabolism and matrix effect were not taken into account. This is due to the fact that in vitro approaches enable the specific mechanisms of action of each molecule/group of molecules to be identified, with relatively low costs. Unfortunately, in vitro studies do not take into account the metabolic transformations and physiological concentrations [5].

As regards in vivo studies, although several epidemiological and clinical studies have evaluated the polyphenol intake, several of them present several limitations, such as a low number of participants, no controls, the use of different methodologies to assess dietary habits, heterogeneous types of database to determine the consumption, etc. Despite the fact that other large and well-designed studies (for example, the PREDIMED study) have shown that the Mediterranean diet, characterized by foods rich in polyphenols, is associated with a reduction in cardiovascular risk and a better cognitive function in the elderly, strong evidence supporting its effects on human health is still not clear [6]. This is also due to the scarcity of knowledge regarding polyphenol bioavailability, together with the difficulty in determining which specific molecule is involved in the biological effect when different phenol compounds are present concomitantly. To date, only cocoa and extra virgin olive oil have thus received the approval of a health claim related to the content in phenol compounds.

Lastly, besides the polyphenol content in foods, in order to establish a correlation between the bioavailability and health effects, their mean intake in humans has to be considered. In a recent systematic literature revision, including more than 90 human studies, the polyphenol intake was estimated according to different dietary habits. Total polyphenol intake for the general population (inclusive of young people, adults, and the elderly) was estimated to be 0.9 g per day, where the main dietary founts were coffee, tea, wine (especially red wine), fruits, and vegetables. Total flavonoids and specific subclasses (flavonols, anthocyanidins, proanthocyanidins, flavan-3-ols, flavones, and flavanones) were associated with reduced CVD, diabetes mellitus (T2D), and mortality for all causes [7]. However, a correlation with the bioavailability of these compounds was not evaluated.

The goal of the present review was to assess the human bioavailability of the main classes of polyphenols, taking into account their food sources and the main factors affecting their in vivo accessibility.

## 2. Materials and Methods 

The most important life-science databases of references and abstracts (PubMed, MEDLINE, Embase, and CAB-Abstract) were methodically analyzed (from database beginning to October 2020), using the following terms: “polyphenols”, “anthocyanins”, “flavanols”, “flavonols”, “flavans”, “stilbenes”, “flavanones” in combination with “bioavailability”, “disease”, “health” and refining the results for “human studies” AND “controlled trials”.

A search by title and abstract led to the collection of 98 relevant publications on the association between polyphenols and bioavailability. By removing duplicates and non-relevant papers (papers investigating the effects of polyphenols without considering bioavailability data, studies performed on animals, or using in vitro models), in total, 37 publications were included in this review. 

## 3. Results and Discussion

### 3.1. Anthocyanins

Anthocyanins are pigments soluble in water contributing to the red, violet, and blue colors in flowers and fruits. At low pH, the anthocyanin chemical structure presents a positive charge at the oxygen atom of the C-ring, called the flavylium ion, and appears as red pigments. Anthocyanins are classified on the basis of the number and position of -OH groups on the flavonoid molecule. To date, more than 600 anthocyanin compounds have been identified [2]. Of these, the glycosylated form of cyanidin, delphinidin, malvidin, peonidin, petunidin, and pelargonidin are the most abundant. The most common sugar in anthocyanidin glycosides is glucose; however, rhamnose, galactose, and rutinose may also be present. The sugar group can be acylated, generally at C3 position, by aromatic acids such as hydroxycinnamic acids (ferulic, caffeic, and coumaric). 

Anthocyanins are among the polyphenol compounds with the highest concentration in foods, with an average concentration of 115 ± 259 mg 100 g^−1^ [8]. The richest sources of anthocyanins (as glycosides) are black elderberries (1316 mg 100 g^−1^), black chokeberry (878 mg 100 g^−1^), and black currant (595 mg 100 g^−1^). Apart from red fruits, important sources of anthocyanins are represented by red wine, colored beans, and vegetables such as red oranges, red lettuce, or red onions. Like other flavonoids, the mean daily intake of anthocyanins can vary among countries, depending on the nutritional habits and cultural differences. It has been calculated that the daily intake of anthocyanins is between 6.8 mg per day in Brazil (where the most important dietary sources are citrus and tropical fruits) and Australia, and 133 mg per day in Italy (the main dietary sources are seasonal fruits, citrus fruits, leafy vegetables, and wine) [9,10,11]. 

Anthocyanin bioavailability is extremely low: only about 1–2% maintain their original structure after ingestion [12]. Anthocyanins exist in different chemical structures depending on the pH. In the stomach, at pH 1.5–3, the main chemical forms are flavylium cations, while in the intestinal environment, the carbinol forms predominate, with lower absorption. In addition, other biotransformation steps occur during gastrointestinal digestion, such as phase II metabolism processes (glucuronidation, sulphation, and methylation), enzymatic and microbiota catabolism [12]. These lead to several chemical compounds, namely anthocyanin glucuronides, phenolic acids (ferulic acid, caffeic acid, vanillic acid, gallic acid, protocatechuic acid, syringic acid, and 4-hydroxybenzoic acid), and aldehydes (phloroglucinaldehyde and phloroglucinaldehyde) [12]. Nevertheless, there is a remarkable inter- and intra-variability in the bioavailability of anthocyanins, due to several factors, such as the food matrix or technological/processing conditions, enzymatic patterns, and microbiota composition. Only a few studies have evaluated a correlation among the results obtained and the bioavailability data. Table 1 summarizes the human trials where the health properties of anthocyanins are correlated with bioavailability studies. The literature data show that the anthocyanin daily doses used in the clinical trials ranged between 2.1 and 94.47 mg. These amounts were generally provided by food (blackcurrant and orange juice); and only the study by Xie et al. (2017) used a food supplement [13]. When anthocyanins were consumed in food, they did not affect the biomarkers investigated, mainly associated with the cardiovascular function, such as the oxidative status, inflammation and vascular reactivity. Only in the study by Xie et al., (2017) 500 mg/day of Aronia extract (*Aronia melanocarpa*, also known as black chokeberry), containing 45.1 mg anthocyanins, 35.7 mg hydroxycinnamic acids, and 41.9 mg proanthocyanidins, improved the total plasma cholesterol and LDL (Low-Density Lipoproteins) receptor in peripheral blood mononuclear cells [13]. This positive effect was due to the duration of the study (12 weeks vs acute consumption or a maximum of four weeks in the other studies) and by the synergistic effect of other phenol compounds present in the extract (hydrocynnamic acids and proanthocyanidins). These results are also supported by the literature data, where the chronic consumption, from six weeks to two months, of Aronia berry extracts (255–300 mg/day) decreased several biomarkers of cardiometabolic diseases, due to the high Aronia polyphenol content [14,15]. In addition, the studies were generally performed on healthy subjects, thus making it more difficult to measure evident effects on the health status. 

Regarding the bioavailability data, the amounts of anthocyanins found in plasma or urine were generally low, which was correlated with the dose taken and the kind of food provided in the studies. Jin et al. (2011) hypothesized that blackcurrant juice did not ameliorate the vascular reactivity in healthy subjects, due to the low levels of anthocyanins in the juice (20%) [16]. This observation has been supported by other authors, who reported that a consistent elevation of plasma anthocyanins and, as a consequence, significative health effects, can be observed only when anthocyanins are consumed at pharmacological levels (500–1500 mg/day) [17,18]. It is also noticeable that anthocyanin compounds are affected by high instability and susceptibility to degradation, particularly at the gastrointestinal level [19]. In addition, the short periods of supplementation or juice ingestion (maximum 12 weeks) were also found to influence the lack of a significant reduction in oxidative status and CVD biomarkers. In urine, the percentage of anthocyanins varied between 0.009 ± 0.002 and 0.79 ± 0.90% of the dose taken, in line with the literature data [20,21]. Despite the great variability in anthocyanin food content, cyanidin and peonidin glucosides were generally considered the most available, since their metabolites were the only ones measurable in plasma or urine. In their clinical trial, Xie et al. (2017) speculated that the 8% reduction in fasting plasma total cholesterol after 12 weeks of supplementation with Aronia extract was also due to cyanidin methylated metabolite and 3-(4-hydroxyphenyl)propionic acid derived from microbiota fermentation [13]. However, the mechanism by which these metabolites affect lipid metabolism needs to be further explored. Despite the general lack of positive effects on CVD biomarkers, several trials have described positive effects of anthocyanins when included in the daily diet. For example, Wedick et al. (2012) reported that the anthocyanin intake of about 22.3 mg per day and an anthocyanin-rich fruit consumption (≥5 times per week) was correlated with a minor risk of developing type-2 diabetes [22]. Cassidy et al. (2016) reported a decreased risk of myocardial infarction in normotensive patients > 65 years, when high levels of anthocyanins were consumed (>35 mg/day) [23]. Other studies reported positive effects of food supplements containing anthocyanins on ocular function, showing encouraging results in relation to glaucoma and in the reduction of retinal oxidative stress due to aging. However, in these studies, anthocyanin bioavailability was not considered. Manach et al. (2005) partially explained the discrepancies in the positive effects of anthocyanin despite their low anthocyanin bioavailability, by considering the following: (1) the possible presence of anthocyanin metabolites not measured in biological samples, such as microbiota metabolites; (2) the instability of anthocyanin metabolites (glucuronides and sulfates), which can extensively degrade in frozen urine, during storage [20].

### 3.2. Flavanols

Flavanol compounds are included in a wide of range of foods. The main sources of flavanols are cocoa (3411 mg 100 g^−1^) and dark chocolate (1590 mg 100 g^−1^). Berries are also an important source of flavanols, containing 659, 330, and 139 mg 100 g^−1^ for black chokeberry, blueberry, and blackcurrant, respectively. Strawberry (148 mg 100 g^−1^) and apple (111 mg 100 g^−1^), as well as hazelnut, pecan nut, pistachio, and almonds (181–496 mg 100 g^−1^), are other important sources. Black tea, green tea, and red wine contain high levels of flavanols, in particular, catechins and proanthocyanidin dimers, with estimated mean amounts of 18–50 mg in the daily diet [20]. 

However, the content of some flavanols is often underestimated, since generally the methods used (e.g., HPLC) only evaluate monomers and proanthocyanidin dimers and trimers. The intake of flavanols in the European Union, according to the EFSA (European Food Safety Authority) Comprehensive European Food Consumption Database, and the average intake of flavan-3-ol monomers, theaflavins, and proanthocyanidins ranges from 181 mg per day (Czech Republic) to 793 mg per day (Ireland) [28].

The highest intakes of flavan-3-ols (monomers) and theaflavins were detected in Ireland (191 and 505 mg per day, respectively) and the lowest in Spain (24 and 9 mg per day, respectively). On the other hand, the highest daily intake of proanthocyanidins (PA) was found in Spain (175 mg per day) and lowest in The Netherlands (96 mg per day). The most important sources were tea (62%), pome fruits (such as apples and pears) (11%), berries (3%), and cocoa derivatives (3%). Tea was the principal contributor to monomer intake (75%), followed by pome fruits (6%). In addition, pome fruits were the most important source of proanthocyanidins (28%). Table 2 reports the clinical trials where the flavanol intakes and their bioavailability were correlated with beneficial effects. Flavanol intake ranged between 28.3 to 907.5 mg/day. These amounts were chosen on the basis of the mean dietary intake of flavonosl among the population included in the studies, and thei consumption with food (enriched chocolate or coffee). Participants in the studies were generally healthy or had stage 1 hypertension without concomitant risk factors. 

Dower et al. (2016) compared the chocolate consumption (containing 150 mg epicatechin, EC) with pure epicatechin supplementation (100 mg) [29]. The length of treatment varied between acute consumption to 18 weeks. The principal outcomes investigated were associated with cardiovascular function (e.g., blood pressure, flow-mediated dilation, and platelet function) and antioxidant activity (e.g., LDL oxidation or plasma 8-isoprostane). A significant improvement in cardiovascular biomarkers was also generally observed for the consumption of low levels of flavanols (28.3 mg/day). A significant improvement in cardiovascular biomarkers was also generally observed for the consumption of low levels of flavanols (28.3 mg/day), despite the EFSA highlighting of a cause-effect correlation between cocoa consumption and endothelium vasodilation for a daily intake of 200 mg [30]. In contrast, only a few studies have measured notable effects on oxidative stress, suggesting that other mechanisms can be involved in the improvement of cardiovascular function [31,32].

According to Taubert et al. (2007), the positive results were generally associated with the monomers epicatechin/catechin and the dimers procyanidin B2/procyanidin B2 gallate, which were the only monomers dosed in plasma, at a concentration ranging between 0.14 (±0.06) ng/mL of procyanidin B2 gallate and 3.63 (±1.02) ng/mL (from 1 to 6 h after consumption) [32]. These amounts reduced blood pressure by 1.9 (±1) mmHg in patients with stage 1 hypertension probably due to the “chronic” increase in endothelium NO production. Similar effects were observed in other studies where higher levels of flavan-3-ols were used, due to the shorter period of intake. Interestingly, in the clinical trial by Dower et al. (2016), the positive effect of epicatechin (EC) on flow-mediated dilatation (FMD) from dark chocolate was not observed for pure epicatechin administered in association with white chocolate, suggesting a significant impact of the food matrix and sugar content on epicatechin bioavailability [29]. 

Urinary flavan-3-ols were measured only by Ostertag et al. (2013), who described a maximum catechin excretion of 13.4 mmol/mol creatinine 2–6 h after dark chocolate intake (containing 907.5 mg total catechins) [33]. As regards platelet function, flavan-3-ols affected platelet aggregation 120 min after intake, when a peak plasma concentration was obtained. However, bleeding time was affected only after 360 min, when the colonic metabolites kicked in Reference [33]. Interestingly, a gender-specific modulation of platelet aggregation reduction was also observed, probably due the formation of larger platelet aggregates after adenosine diphosphate (ADP) stimulation in women. It should also be noted that flavan-3-ol bioavailability varies markedly among the different subclasses. Manach et al. (2005) reported that galloylated catechins are poorly absorbed, explaining the higher bioavailability and the activity of epicatechin described in the clinical trials [20]. In addition, epicatechin glucuronide and sulfate metabolites, together with valerolactone microbial derivatives—not measured in the clinical trials included in this review—account for 6–39% of the ingested epicatechin, thus prolonging its biological effects [34]. 

As regards procyanidins, these compounds have a reduced bioavailability, which is about 100-fold lower than monomers. The biological effects are generally due to the monomers formed after gastric degradation, which can be rapidly absorbed in the gut. In addition, the gut microbiota is responsible for the metabolite formation (m-hydroxyphenylpropionic acid, m-hydroxyphenylacetic acid, phenylpropionic acid, phenylacetic acid, hydroxyphenylvaleric acid, and benzoic acid among others), which could also be responsible for various biological effects [20].

### 3.3. Flavonols

Fruits, vegetables, and some beverages, like tea and red wine, are the main source of flavonols, for which the intake is estimated between 18 (USA) and 58 mg (Japan) per day [28]. However, these intake levels generally refer only to the three main flavonols, namely quercetin, myricetin, and kaempferol. In fruits and vegetables, the highest quercetin content is found in cranberry (149 mg/100 g) and onions (65 mg 100 g^−1^), while in green tea and red wine, the mean contents are 2.5 and 1.6 mg/100 mL, respectively [36]. Eleven studies have explored the different health effects of flavonol intake, mainly on the reduction in CVD risk factors (homocysteine and LDL-oxidation levels, homocysteine, plasmatic High Density Lipoproteins (HDL) and LDL cholesterol, blood pressure, NO production, and platelet aggregation), inflammatory biomarkers (C-reactive protein and endothelin-1 expression), and antioxidant activity (e.g., excretion of urinary F2-isoprostanes and plasma malondialdehyde (MDA)) (Table 3). One study also investigated the effect of an enzymatically modified quercetin on cognitive function [37]. These effects have generally been measured in healthy subjects, apart from three studies involving subjects at cardiovascular risk [37,38,39]. 

In most studies, flavonols were administered by extracts, given alone or mixed with food; only in three studies they were provided using food preparations (onion soup or cake) [40,41,42]. Flavonol intake ranged between 16.7 to 400 mg/day and included mainly quercetin and its derivatives, isorhamnetin and kaempferol. The data in Table 3 show that cardiovascular parameters, as well as oxidative stress biomarkers, were generally not affected by flavonols. Suomela et al. (2006) and Larmo et al. (2009) used a sea buckthorn extract (*Hippophae rhamnoides* L.) as the source of flavonols (78 and 16.7 mg per day, respectively), administered with meals [38,43]. Both flavonol intakes failed to reduce oxidative stress, total and LDL cholesterol, or C reactive protein (CRP) concentration. Sea buckthorn was administered because of its traditional use in Eastern countries and the encouraging results from clinical and epidemiological studies reporting a reduction in cardiovascular risk factors [44]. The authors explained the different outcomes of their studies, using a moderate berry dose, similar to the average consumption in the daily diet and to the dosage (3–9 g) prescribed by the Chinese Pharmacopoeia for ameliorating hematic circulation [45]. These amounts were lower than those used in other trials (600 mg sea buckthorn flavonols), where positive results were reported [46].

Despite the negative results, plasma flavonols (quercetin, kaempferol and isorhamnetin glucuronides, and sulfates) significantly increased after the treatment, with respect to placebo (*p* < 0.005). This means that the initial dose taken correlates closely with the biological effects but is not always explained by the bioavailability of flavonols and the duration of intake. In fact, although the flavonol plasma levels were always measurable, they were not sufficient to affect specific biomarkers. Quercetin, especially in its glucosidic form, is generally efficiently absorbed and bioavailable. In addition, quercetin metabolite elimination is quite low (t_1/2_ 11–28 h), leading to their accumulation in plasma with repeated intakes [20]. Positive results were obtained on FMD and platelet aggregation. 

Bondonno et al. (2016) observed no improvement in blood pressure or FMD in healthy subjects, when increasing amounts of quercetin-3-O-glucoside (50–400 mg/day) were provided [47], but only when quercetin was enzymatically modified to obtain a more bioavailable α-oligoglucosylated quercetin derivative, isoquercitrin. Supplementation with 4.89 mg of this compound significantly increased FMD response by 1.8% compared with the placebo (*p* = 0.025), but with different mechanisms not involving NO production increase. In contrast, cognitive function was not affected. The different form of quercetin used makes it difficult to compare it with other studies; however, the maximum plasma concentration reached 144 ± 12.3 nM, which is higher than the values measured in other studies where quercetin was administered [48,49]. 

Perez et al. (2014) postulated that the vasodilator effects can be mediated by quercetin-3-O-glucuronide (Q3GA) deconjugation mediated by plasmatic glucuronidase [50]. This is because a plasmatic dose-dependent increase in this metabolite was detected after 200 and 400 mg supplementation with quercetin, which was not detected with other metabolites (quercetin aglycone, isorhamnetin aglycone, and their glucuronide forms). The enzyme glucuronidase is present in lysosomes involved in the glycosaminoglycans cleavage. An extensive inter-individual variability in the activity of glucuronidase has also been described, which may be attributed by variations in its gene sequence or expression. This enzyme hydrolyzes glucuronidated metabolites at the vascular level, producing the parent aglycone, which, due to its elevated liposolubility, accumulates in tissues and performing its biological activity. The effect on arterial diameter was not associated with either the early changes flavonoid plasmatic levels or the glucuronidase activity. Nevertheless, these effects were associated with a combination of both factors. Quercetin provided by food (high-quercetin onion soup) or supplements (providing 138 and 150 mg quercetin, respectively) was shown to be effective in inhibiting platelet aggregation mediated by collagen. The inhibition correlated highly with the AUC of the quercetin metabolites, isorhamnetin and tamarixetin (4-O-methyl-quercetin) [40,48].

### 3.4. Phenolic Acids

Phenolic acids are a class of secondary metabolites, highly distributed among plants. According to their chemical structure, phenolic acids can be divided in benzoic and cinnamic acids. The main benzoic groups are gallic, protocatechuic, and *p*-hydroxybenzoic acids, mainly as conjugates. The highest concentration (fresh weight) of benzoic acids has been calculated in Apiaceae species (spices and herbs): anise 730–1080 mg kg^−1^, cumin up to 42 mg kg^−1^, fennel up to 106 mg kg^−1^, and parsley up to 30 mg kg^−1^ [51,52]. Cinnamic acids are widely distributed in plants, as esters or amides. The most representative are caffeic, chlorogenic, and ferulic acids. High concentrations of cinnamic acids are coffee, tea, wine, cocoa, fruits, vegetables, and cereals. Some of the most important sources of caffeic acid are wild blueberry (1470 mg kg^−1^), coffee (870 mg kg^−1^), carrots (260 mg kg^−1^), plum (234 mg kg^−1^), and eggplant (210 mg kg^−1^). 

One of the most important derivatives of caffeic acid is caftaric acid, a representative polyphenol in wine (6–73 mg L^−1^ in white wine, 46–141 mg L^−1^ in red wine), while chlorogenic acid is present in considerable levels in coffee (depending on the climatic and processing conditions, and procedures for coffee preparation) [53,54]. The chlorogenic acid content in roasted coffee beans varies depending on the roasting extent, in the range of 2.3–80 g/kg (dried weight) and 890–8130 mg/L in espresso coffee [53]. The intake of chlorogenic acid can be very high; it has been estimated to be up to 0.8 g per day among coffee drinkers [20]. 

Cereals are the most important source of ferulic acid, derivative of cinnamic acid derivative (for which the intake ranges from about 0.092 to 0.32 g) [49]. Table 4 shows the studies correlating phenolic acid intake, their bioavailability, and different health effects, mainly focused on blood pressure, vasodilation, antioxidant activity, and inflammation. Six of ten studies included in this review investigated the effects of chlorogenic acid and its metabolites in coffee or in beverages prepared in order to mimic coffee intake; one study used purified caffeoylquinic acid (5-CQA); one study included whole grain; and two studies included a blueberry drink. Phenolic acids were administered to healthy subjects in the range of 138.7 and 900 mg/day and were tested both in acute and chronic consumption (maximum eight weeks). Generally speaking, vascular function was positively affected by chlorogenic acid (CGA) provided by decaffeinated coffee intake (50 mL) or purified caffeoylquinic acid (5-CQA), confirming previous studies showing that phenolic compounds, other than caffeine, can contribute to vasoactive efficacy [55,56]. Observational studies indicate that moderate coffee intake (4 cups), containing from 105 to 500 mg of CGA, is associated with a lower CVD risk [57]. 

Potential mechanisms by which CGA and its main plasma metabolites (5-cholorgenic acid, ferulic-4′ -O-sulfate, and isoferulic-3′-O-glucuronide) mediate the vascular effects include the inhibition of NAPDH oxidase, leading to a reduction in superoxide production and, as a consequence, to an increase in endothelium NO bioavailability [58]. Conflicting positions have been taken regarding the possible agonistic or antagonist effects of caffeine on vascular function. Agudelo-Ochoa et al. (2016), postulated that caffeine could be responsible for the negative effects observed after 400 mL coffee intake (caffeine content < 300 mg), since it can interfere with the mechanism of action of chlorogenic acid, thus decreasing NO production [59]. In contrast, Boon et al. (2017) noticed a vasodilator effect only in subjects consuming caffeinated coffee (270 mg caffeine) but not decaffeinated coffee, although the CGA levels were comparable (300 and 287 mg CGA, respectively) [60]. In plasma, an increase in CGA metabolites (5-CGA) was always measured, even when no significant effects on vascular function were observed, suggesting that synergistic effects on different polyphenol compounds can occur. Rodriguez-Mateos et al. (2013) also observed an amelioration of endothelial function after the acute intake of blueberry drinks containing different levels of polyphenols, from 766 to 1791 mg [61]. Phenolic acid metabolites (caffeic acid, ferulic acid, iso-ferulic acid, vanillic acid, benzoic acid, and 2-hydroxybenzoic acid) were the only ones measured in plasma, while no flavonols or anthocyanins were detected, thus suggesting that these compounds were not responsible for the positive effects noticed. The intake of phenolic acids with whole grains (138 mg/70 g) for eight weeks was associated with a reduced inflammatory status in overweight subjects by Vitaglione et al. (2015), compared with equal amounts of refined wheat (2.6 mg ferulic acid) [62]. 

Considering the bioavailability data, this effect was mainly associated with ferulic acid, whose concentrations were two-fold higher in the feces of subjects consuming whole grains. Interestingly, this result was explained by the fact that the release of FA in the colon could be due to wheat bran polysaccharide fermentation and mediated by bacterial enzymes xylanase and ferulic acid esterase. These enzymes are mainly synthetized by Lactobacilli (Firmicutes), Bifidobacteria (Actinobacteria), Bacteroides, and Prevotella (Bacteroidetes) when arabinoxylans with esterified ferulic acids are introduced [66,67]. Since overweight or obese individuals show reduced amounts of Bacteroidetes and Bifidobacteriales, Firmicutes are considered the main responsible for the fermentation of whole-grain polysaccharides and ferulic acid liberation [62,66]. 

### 3.5. Stilbenes, Isoflavones, and Flavanones

Only a few studies were found correlating the effects of stilbenes (resveratrol), isoflavones, and flavanones with their bioavailability (Table 5). Resveratrol is a phytoalexin that is widely distributed in the plant kingdom. It is found in more than 70 species, but grapes and wine are the most important sources. The mean levels of total resveratrol in red wine is 7 mg L^−1^; rose wine has a total of 2 mg L^−1^, and white wine has and 0.5 mg L^−1^ [60]. Resveratrol supplementation (250–500 mg/day for 7 and 28 days, respectively) was investigated in healthy subjects as regards cognitive function and cerebral ematic circulation. Despite total resveratrol metabolites (resveratrol 4′glucuronide, 3′ glucuronide, and sulfate) being ten-fold higher in the treatment group, supplementation failed to improve cognitive function but increased cerebral flow and reduced fatigue levels [68,69]. These effects could also be mediated by unmetabolized resveratrol, since the literature data indicate that it can be found in plasma bound to albumin or LDL, and that it elicits its biological function after interaction with cells that have receptors for albumin and LDL [70]. In brain, resveratrol contributed to vasorelaxation, oxygenation, and sirtuin (SIRT)-mediated increases in mitochondrial gene expression in brain [68]. 

The only source of isoflavones are products derived from soybeans. Depending on the kind of soy preparation, isoflavones can be present as aglycones or glycosides. One study evaluated the impact of isoflavones on triglycerides and oxidative biomarkers [71]. Only two studies investigated the effects of isoflavones (supplemented for 12 months at doses of 60 and 96 mg isoflavone aglycones/day, respectively) on bone density in post-menopausal women with contradictory results. In the study by Lambert et al. (2017), the positive effects were explained by the use of lactic acid probiotic bacteria in the treatment group in association with isoflavones, which mediated equol production [72]. Equol is a derivative of daidzein produced by anaerobic bacteria with great estrogenic potential. Since none of the subjects was able to produce equol at the beginning of the study and, after six months of treatment, 55% of individuals in the Red Clover Extract (RCE) group produced equol, it is plausible that that the probiotics positively affected the participant intestinal bacterial pattern, promoting more positive conditions for equol production. This hypothesis seems to be confirmed by the fact that high plasma levels were found in both studies; however, in the study by Brink et al. (2008), bone density was not affected by isoflavones [73]. However, a higher number of studies is necessary to confirm these results.

Only one study investigated the effect of flavanones, in particular hesperidin on vascular function, providing 320 mg with 767 mL orange juice/day as acute consumption. Despite the detection of several hesperidin metabolites in plasma (hesperidin-glucuronide, naringenin-7-O-glucuronide, dihydroferulic acid, dihydroferulic acid–glucuronide, hippuric acid, and vanillic acid 4-hydroxyphenylacetic acid) after 5 h, no significant effects were measured. Despite previous studies showing that plasma hesperidin metabolites were correlated with health effects on the endothelium after flavanone acute consumption [75,76], further investigations are necessary to evaluate and support the biological effects of these polyphenols.

## 4. Conclusions

The data reported in this review highlight that, despite the very large number of studies investigating the health effects of polyphenols in humans, only a few have considered their bioavailability in order to partially support the associated bio-efficacy. The bioavailability of polyphenols varies among the different classes and ranks as follows: phenolic acids > isoflavones > flavonols > catechins > flavanones, proanthocyanidins > anthocyanins, confirming data from previous pharmacokinetic studies [20]. Apart from favanols and flavonols, the amounts of polyphenols used in studies were considered to be too low to reach significant plasma concentrations that would provide beneficial effects. However, the amounts were chosen on the basis of the mean consumption in the daily diet of the populations included in the studies. Another point to be considered is that healthy subjects were mainly included in clinical trials. On the one hand, it makes it more difficult to measure significant changes in biomarkers generally associated with pathological conditions; on the other hand, it suggests a potential role of polyphenols provided with the daily diet or supplementation in maintaining the health status. In fact, positive variations of physiological parameters generated by polyphenol intake could help in improving or modulating specific functions and reducing some risk factors for chronic diseases. Cardiovascular function was the main health area investigated. Vasodilator effects were found for phenolic acids (mainly chlorogenic acid and ferulic acid) and flavanols (in particular catechins and proanthocyanidins), which were partially explained by their bioavailability. 

As regards anthocyanins, their plasmatic levels were too low to affect the biomarkers considered; however, cyanidin and peonidin were the most available. Future research should focus on confirming and integrating the data discussed in this review, particularly for stilbenes, isoflavones, and flavanones, since few studies have associated their bioavailability with health effects. In addition, the biological effects of phenol metabolites derived from microbiota fermentation should be more extensively studied, since several data suggest their role in mediating the benefits of polyphenols.

Finally, since polyphenol bioavailability can be affected by food matrix components, specific strategies could be considered in order to increase their in vivo delivery (e.g., fermentation or exploiting the association among foods), or to protect them from degradation (e.g., microencapsulation).

## Figures and Tables

**Table 1:** Anthocyanin’s beneficial effects and bioavailability in human subjects.

Cohort and Study Details	Anthocyanin Intake	Aim	Bioavailability Data	Outcome	Reference
49 healthy adult former smokers (25 M; 24 F) (mean age 35 ± 2.8 years) Duration: 12 weeks Randomized controlled trial	Treatment group (*n* = 25): 500 mg *Aronia melanocarpa* extract (9.02% anthocyanins, 7.14% hydroxycinnamic acids and 8.38% proanthocyanidins). Placebo group (*n* = 24): 500 mg rice powder with 0.2% beet juice concentrate.	Modulation plasma lipids (change in LDL cholesterol was the primary outcome), blood pressure, biomarkers of inflammation and oxidative stress, lipid transport genes of peripheral blood mononuclear cells.	Overnight urinary anthocyanins were significantly higher in the treatment group vs. placebo group (0.332 ± 0.136 vs. 0.051 ± 0.022 mg mg^−1^ creatinine). Urinary peonidin-3-galactoside was 0.0062 ± 0.0026 mg mg^−1^ creatinine in the treatment group vs. 0.0008 ± 0.0005 mg mg^−1^ creatinine in placebo group (*p* < 0.05). The excretion of the other polyphenols was not significantly affected.	After 12 weeks, Aronia consumption compared with the placebo group showed: ↓ 8% fasting plasma total cholesterol (*p* = 0.0140), ↓ 11% LDL cholesterol (*p* = 0.02), ↓ LDL receptor protein in peripheral blood mononuclear cells (*p* = 0.0036).	[13]
20 Healthy subjects (UK) (9 M; 11 F) mean age (44.55 ± 13.34 years) Duration: acute consumption Randomized, double-blind, placebo-controlled crossover study	250 mL of either a blackcurrant juice drink (20% of anthocyanins) or the control drink. Sample collection after consumption: Blood: periodically up to 480 min; Urine: every 2 h and at 24 h.	To measure vascular reactivity at 120 min after juice consumption.	The urinary percentage anthocyanins excreted after 120 min was 0.021 ± 0.003% and 0.009 ± 0.002% of the dietary intake of delphinidin glycosides and cyanidin glycosides, respectively.	No significant effects on vascular reactivity were found. An increase in plasmatic vitamin C was observed (*p* = 0.006).	[16]
15 subjects with coronary artery disease (13 M; 2 F) (mean age: 62 ± 8 years) Duration: acute consumption Pilot study	480 mL cranberry juice (54% juice; 835 mg total polyphenols; 94.47 mg anthocyanins). Sample collection after consumption: Plasma and urine: after 4 h.	To evaluate plasma redox capacity.	Plasma concentrations ranged between 0.56 and 4.64 nmol L^−1^. Total urinary anthocyanins were 0.79 ± 0.90% of the amount taken. Cyanidin-glucoside and peonidin-glucoside were the most available (0.007 ± 0.004% and 0.029 ± 0.059% of the dose delivered).	Anthocyanin plasmatic concentrations were not able to reduce oxidative stress.	[24]
20 healthy females (mean age: 27.8 ± 7 years) Randomized controlled trial Duration: 2 weeks	Treatment group (*n* = 11) 750 mL per day (3 × 250 mL) of cranberry juice (total anthocyanins = 2.80 ± 0.19 mg L^−1^; where 29.2% peonidin galactoside, 26.1% cyanidin arabinoside, 21.7% cyanidin galactoside, 17.5% peonidin arabinoside, 4.1% peonidin glucoside, and 1.4% cyanidin glucoside). Placebo group (*n* = 9): natural mineral water with strawberry flavor.	Plasma antioxidant activity and biomarkers of oxidative stress (total phenol concentrations, reduced glutathione levels (GSH) and plasma free radical trapping capacity (FRAP)). Activity of blood’s antioxidant enzymes (SOD, catalase and GSH-Px). Urinary excretion of MDA and 8-oxo-deoxyguanosine (as marker of DNA damage).	Neither anthocyanins nor catechins were detectable in plasma samples isolated from both groups, only vitamin C increased significantly (*p* < 0.01) in the cranberry juice group. Further, catechins were not detectable in the urine samples.	Hcy, TC, TG, HDL, and LDL were unchanged. The antioxidant potential of the plasma, GSH-Px, catalase and SOD activities, MDA and 8-oxo-deoxyguanosine levels were not significantly different in both groups.	[25]
18 healthy subjects (10 M; 8 F) (25–47 years) Duration: 4 weeks Randomized crossover study	1 L per day of either blood orange juice (OJ) (from Moro, Tarocco, and Sanguinello varieties) or blond OJ that contains no anthocyanins (from Valencia, navel, and Belladonna varieties). Blood orange juice contained: 53.09 ± 5.31 mg L^−1^ total anthocyanins, 3.96 ± 0.20 mg L^−1^ delphinidin-3-glucoside, 25.79 ± 1.17 mg L^−1^ cyandin-3-glucoside, 17.88 ± 0.95 mg L^−1^ cyanidin-3-(6-malonylglucoside).	Potential effects on cell markers of platelet and leukocyte activation (P-selectin, PAC-1, leukocyte activation markers CD11b) due to anthocyanins absorption after daily supplementation with blood OJ for 1 month.	Mean levels of anthocyanins (11.47 ± 5.63 nmol L^−1^) significantly differed from baseline in 24-h urinary excretion. Anthocyanins levels remained substantially unchanged until the end of treatment (*p* = 0.1).	The anthocyanin plasma levels reached were insufficient to significantly modify cell markers of platelet and leukocyte activation and interaction. Urinary excretions of anthocyanins considered showed a significant increase after blood OJ consumption (*p* < 0.05).	[26]
16 healthy females (20–27 years) Duration: 3 weeks Randomized crossover study	Group A (*n* = 8) - Standardized diet without orange juice; - Wash out period; - Standardized diet with 600 mL/day of blood orange juice. Group B (*n* = 8) - Standardized diet with 600 mL/day of blood orange juice; - Wash-out period; - Standardized diet without orange juice. Each period was of 21 days. 100 mL of orange juice contained: 75.2 mg vitamin C, 67 μg of β-cryptoxanthin, 20 μg of lutein, 18 μg of zeaxanthin, 17 μg of lycopene, 10 μg of β-carotene, and 8 μg of α-carotene, 3.5 mg cyanidin-3-glucoside, 1.2 mg cyanidin-3-glucoside-6″-malonyl.	To evaluate the effect on plasma antioxidant concentrations and on markers of lipid peroxidation malondialdehyde (MDA) and 11-dehydro-tromboxane 2 (TXB2)	Cyanidin 3-glucoside mean plasma concentration was, after the washout period, about 0.6 nmol L^−1^ and increased from 0.56 nmol L^−1^ to ∼8 nmol L^−1^ after 3 weeks of blood orange juice intake (*p* < 0.05). Both the aglycone and the cyanidin-3-glucoside-6″-malonyl were not detected in plasma.	Plasma antioxidant capacity did not increase after the 3 weeks of juice intake. The daily intake of orange juice did not affect the biomarkers of lipid oxidation malondialdehyde (MDA), and 11-dehydro-TXB2.	[27]

LDL, Low Density Lipoproteins; GSH-Px, glutathione peroxidase; MDA, malondialdehyde; PAC-1, procaspase-activating compound-1; SOD, superoxide dismutase; OJ, orange juice; Hcy, Homocysteine; TG, triglycerides.

**Table 2:** Flavanols’ beneficial effects and bioavailability in human subjects.

Cohort and Study Details	Flavanol Intake	Aim	Bioavailability Data	Outcome	Reference
20 healthy men (40–80 years) Duration: acute consumption randomized, placebo controlled, crossover study	Subjects consumed the following: Dark chocolate (DC) (70 g, containing 0.15 g EC) associated with 2 capsules of placebo; Pure EC (0.050 g, purity 96.2%) (total EC = 0.100 g) associated with white chocolate (WC) (75 g); 2 capsules of placebo with 75 g of WC (without EC).	To study the effect of epicatechin from different matrices (cocoa and supplement) on vascular function (FMD and AIx).	DC intake determined an Area Under the Curve of 16.2 μM (per 0.100 g of EC) that was not significantly different from Area Under the Curve calculated for pure EC (*p* = 0.14).	Pure EC didn’t ameliorate Flow Mediated Dilatation (+0.75%; *p* = 0.10) or Augmentation Index (2.2%; *p* = 0.23) respect to placebo. DC significantly ameliorated Flow Mediated Dilatation (+0.96%; *p* = 0.04) and Augmentation Index (4.6%; *p* = 0.02). Amelioration of FMD (+0.21%; *p* = 0.65) or Aix (–2.4%; *p* = 0.20) was not different after pure EC and DC intake. EC bioavailability was not different after pure EC and DC (*p* = 0.14).	[29]
21 healthy subjects (11 M; 11 F) (mean age: 32.2 ± 3.1 years) Duration: 2 weeks Randomized, double-blind, controlled with placebo study	The following treatments were assigned: High-flavonoid chocolate intake (0.213 g PC, 0.046 g EC) per day; Low-flavonoid DC (46 g) per day.	To evaluate the impact of DC enriched with flavonoids on FMD, blood pressure, markers of oxidative stress (LDL-ox, total antioxidant power, 8-isoprostane levels and plasmatic lipid concentrations).	Plasmatic EC levels were significantly higher at two weeks after high-flavonoid DC consumption (204.4 ± 18.5 nmol L^−1^, *p* < or =0.001) than after low-flavonoid DC intake (17.5 ± 9 nmol L^−1^, *p* = 0.99).	The intake of chocolate containing high levels of flavonoids ameliorated FMD (average change = 1.3 ± 0.7%) respect to the groups consuming chocolate with low levels of flavonoids (mean change = −0.96 ± 0.5%) (*p* = 0.024). LDL-ox, antioxidant power, 8-isoprostanes, blood pressure, lipid biomarkers were not significantly in the two groups.	[31]
44 adults (20 M; 24 F, 20) (Age: 63.6 ± 4.8 years) with not-treated hypertension (at stage 1) without other diseases Duration: 18 weeks Randomized study	Treated subjects (*n* = 22): 6.3 g dark chocolate per day providing 30 mg of polyphenols per day (1.4 mg gallic acid, 1.7 mg catechin, 5.1 mg epicatechin, 0.3 mg epicatechin-gallate, 6.8 mg procyanidin dimer, 1.8 procyanidin dimer-gallate, 5.3 mg procyanidin trimer, 1.0 mg procyanidin trimer-gallate, 3.7 mg procyanidin tetramer, 2.6 mg procyanidin pentamer, <0.05 mg flavonols, 3.1 mg caffeine, 26.4 mg theobromine) Control group (*n* = 22) polyphenol-free white chocolate.	To evaluate the change in blood pressure. Secondary objectives were the assessment of nitric oxide and oxidative stress plasmatic biomarkers (S-nitrosoglutathione and 8-isoprostane, respectively).	Only catechin, epicatechin and the dimers procyanidin B2 and procyanidin B2 gallate were measured in plasma and didn’t change after 18 weeks. Pharmacokinetic data were: Epicatechin: AUC from 761 (±210) ng mL^−1^ × min on day 1 to 774 (±253) ng mL^−1^ × min (*p* = 0.82) at 18 weeks; Cmax from 3.63 (±1.02) ng/mL on day 1 to 3.58 (±0.92) ng mL^−1^ (*p* = 0.78) at 18 weeks; Tmax from 77 (±4) min on day 1 to 81 (±6) min at 18 weeks (*p* = 0.70); T1/2 from 54 (±3) min on day 1 to 56 (±5) min (*p* = 0.79) at 18 weeks. Catechin: AUC from 234 (±61) (day 1) to 228 (±56) ng mL^−1^ × min (*p* = 0.77); Cmax from 1.12 (±0.31) ng mL^−1^ on day 1 to 1.01 (±0.26) ng mL^−1^ (*p* = 0.58) at 18 weeks; Tmax from 78 (±9) min on day 1 to 82 (±6) min at 18 weeks (*p* = 0.69); T1/2 from 54 (±6) min on day 1 to 58 (±7) min (*p* = 0.68) at 18 weeks. Procyanidin B2: AUC from 99 (±30) ng mL^−1^ × min on day 1 to 102 (±32) ng mL^−1^ × min (*p* = 0.90); Cmax from 0.45 (±0.15) ng mL^−1^ on day 1 to 0.43 (±0.14) ng mL^−1^ (*p* = 0.94) at 18 weeks; Tmax from 81 (±8) min on day 1 to 86 (±9) min at 18 weeks (*p* = 0.62); T1/2 from 56 (±6) min on day 1 to 57 (±5) min (*p* = 0.91) at 18 weeks; procyanidin B2 gallate: AUC from 33 (±14) ng mL^−1^ × min on day 1 to 33 (±13) ng mL^−1^ × min (*p* = 0.91); Cmax from 0.14 (±0.06) ng mL^−1^- day 1 to 0.14 (±0.06) ng mL^−1^ (*p* = 0.98) at 18 weeks; Tmax from 89 (±10) min on day 1 to 85 (±8) min at 18 weeks (*p* = 0.72); T1/2 from 62 (±7) min on day 1 to 59 (±6) min (*p* = 0.76) at 18 weeks.	At the end of the study, the treatment with dark chocolate educed mean systolic blood pressure by 2.9 (±1.6) mm Hg (*p* < 0.001) and diastolic blood pressure by 1.9 (±1) mm Hg (*p* < 0.001) but 8-isoprostane levels didn’t change. Hypertension prevalence was reduced from 86% to 68%. S-nitrosoglutathione levels (increased by 0.23 nmol L^−1^ (±0.12) (*p* < 0.001).	[32]
42 healthy subjects (26 M; 16 F) (mean age 41 ± 2.0 years) Duration: acute consumption Observer-blinded randomized-controlled crossover acute intervention trial	Subjects received 60 g of dark chocolate enriched with flavan-3-ols (with 400 mL water), 60 g of a “standard” dark chocolate or 60 g of white chocolate (14 days wash out period was used between treatments). Sixty grams of chocolate enriched with flavan-3-ols contained: 0.257 (±1.06) g epicatechin, 53.6 (±0.27) mg catechin, 0.198 (±1.22) g dimer B2, 0.168 (±1.42) g trimers, 0.105 ± (12.75) g tetramers, 0.125 ± (6.04) g pentamers (total flavonoids = 907.4 ± 22.75 mg). Sixty grams of “standard” dark chocolate contained: 84.1 (±0.67) mg epicatechin, 25.8 (±1.02) mg catechin, 74.4 (±0.76) mg dimer B2, 47 (±2.23) mg trimers, 32.1 (±4.4) mg tetramers, 0.119 (±39.5) g pentamers (total flavonoids: 382.3 ± 45.2 mg). White chocolate = ND.	To evaluate if flavan-3-ol-enriched dark chocolate could influence markers of platelet status (adenosine diphosphate-induced platelet aggregation, expression of P-selectin, thrombin receptor-activating peptide-induced platelet aggregation and thrombin receptor-activating peptide-induced fibrinogen binding, collagen/epinephrine-induced bleeding time).	Plasmatic levels of total C/EC significantly increased 120 min after flavan-3-oil enriched or “standard “dark chocolate in comparison with white chocolate (*p* < 0.001) (Cmax = 1.20 μmol L^−1^ 120 min after consumption of dark chocolate enriched with flavan3-ols) and decreased after 6 h. Urinary levels of total catechins increased 120 and 360 min after consumption of enriched dark or “standard” dark chocolate in comparison with white chocolate (*p* < 0.001), reaching a peak concentration of 13.4 mmol mol^−1^ creatinine. Comparable results were observed for procyanidin dimer B 2 (*p* < 0.001), reaching a peak concentration at 57 μmol mol^−1^ creatinine at 360 min. The peak of flavan-3-ol concentrations was higher in biological fluids of women (*p* = 0.047).	Enriched dark and white chocolate ameliorated different biomarkers of platelet status (adenosine diphosphate-induced platelet aggregation, P-selectin expression, thrombin receptor-activating peptide-induced platelet aggregation and thrombin receptor-activating peptide-induced fibrinogen binding) following a gender-specific fashion.	[33]
18 healthy male subjects (mean age: 36 ± 10 years) Duration: acute consumption Observational study	Subjects consumed 50 g of 90% cocoa chocolate (containing 7.5 g total polyphenols expressed as GA equivalents) within 5 min.	To investigate the impact of dark chocolate on platelet status and the correlation with flavanols metabolites.	After 4 h from the ingestion, epicatechin metabolites concentration in plasma significantly increased (epicatechin glucuronide, sulfate, methyl-epicatechin-sulfate) (*p* < 0.05).	An inhibitory effect of cocoa was observed on platelet aggregation and adhesion caused by ADP/collagen 4 h after cocoa intake (98.5 ± 13.0 respect to 114.5 ± 22 s, *p* < 0.001) was noticed, while the closure time of collagen/epinephrine didn’t change (128.5 ± 27.0 vs. 122.5 ± 34. s, *p* = 0.33), probably associated with EC metabolites.	[35]

ADP, Adenosine diphosphate; AUC, area under the curve; DC, Dark Chocolate; EC, epicatechin; FMD, flow-mediated dilatation; GA, gallic acid; Aix, Augmentation Index; PC, procyanidins; LDL-ox, oxidated LDL.

**Table 3:** Flavonols’ beneficial effects and bioavailability in human subjects.

Cohort and Study Details	Flavonol Intake	Aim	Bioavailability Data	Outcome	Reference
25 participants (12 M; 13 F) (mean age: 64.1 ± 6.3 years) with at least one CVD risk factor (sBP 120–160 mmHg, FPG 5.6–6.5 mM, total cholesterol 5–8 mM or a waist circumference > 94 cm for men or >80 cm for women) Duration: acute consumption Randomized, controlled crossover trial	Treatment group: - 4.89 mg/kg bw/day of EMIQ^®^ (Enzymatically Modified IsoQuercitrin), - half teaspoon of maltodextrin, - one and half tablespoons of Cottee’s Raspberry flavored cordial. Placebo group: - half teaspoon of maltodextrin, - one and half tablespoons of Cottee’s Raspberry flavored cordial, The treatments were given in 250 mL of water.	To evaluate if FMD, BP, and cognitive function improve whether an acute intake of EMIQ^®^ was administered.	After 3 h from the consumption of EMIQ^®^, quercetin metabolites concentration was significantly higher in plasma respect placebo group (quercetin aglycone 144.9 ± 12.3 nM vs. 12.6 ± 12.3 nM; and isorhamnetin 245.5 ± 16.5 nM vs. 41.7 ± 16.5 nM) (*p* < 0.001).	EMIQ^®^ significantly affected FMD compared with the placebo (*p* = 0.025).	[37]
14 healthy males (46.6 ± 5.6 years) with a slightly elevated total cholesterol level (5.3–7.2 mmol/L) Duration: 4 weeks Double-blind, placebo-controlled, crossover study	Group 1: daily consumption of 185 g of an oatmeal porridge supplemented with a flavonols extract of sea buckthorn (*Hippopae rhamnoides* L.) Group 2: consumption of control porridge without flavonols. 0.4 g of extract, added to the porridge, contained: 78 mg of total flavonol aglycones, of which 70% isorhamnetin, 26% quercetin, and 4% kaempferol.	To evaluate the effects on CRP, conjugated dienes and oxidized LDL, homocysteine, and paraoxonase activity (potential risk factors of CVD) of a flavonols extract of sea buckthorn.	Flavonols were mainly present as glucuronide and sulfate metabolites in plasma fluid. When was intake porridge added with flavonols extract, AUC was significantly higher for kaempferol and isorhamnetin (*p* < 0.05).	The flavonols ingested did not significantly affect the following: - Oxidized LDL; - CRP; - Homocysteine levels; - Plasma antioxidant potential; - Paraoxonase activity.	[38]
9 overweight/obese men (*n* = 4) and post-menopausal women (*n* = 5) (mean age = 55.9 ± 2.1 years) Duration: acute consumption Randomized, crossover study	Subjects ingested quercetin aglycone (1095 mg) with 3 types of standardized breakfast: - Fat-free (<0.5 g); - Low-fat (4.0 g); - High-fat (15.4 g).	To verify whether dietary fat improve quercetin and its metabolites bioavailability in adults with high CVD risk.	During the high-fat breakfast, compared to the fat-free trial: - Plasma quercetin: ↑ 45% Cmax; ↑ 32% AUC (0–24 h); - plasma isorhamnetin: ↑ 40% Cmax; ↑ 19% AUC (0–24 h); - Plasma O-methyl-isorhamnetin: ↑ 46% Cmax; ↑ 43% AUC (0–24 h).	Dietary fat improved quercetin bioavailability by increasing its absorption leading to a possible dietary approach for reducing CVD risk.	[39]
6 heathy subjects (4 M; 2 F) (mean age 34 ± 7 years) Duration: 1 day Randomized, double-blind, crossover study	Participants ingested either a high- or a low-quercetin soup (600 mL), made using 500 g of onions for portion. 1 L of low quercetin onion soup (LQS) contained 0.1 mg L^−1^ quercetin aglycone, 3.8 mg L^−1^ quercetin-4′-glucoside, 4.3 mg L^−1^ quercetin 3,4′-glucoside. 1 L of high quercetin onion soup (HQS) contained: 1.1 mg L^−1^ quercetin aglycone, 53.2 mg L^−1^ quercetin-4′-glucoside, 60.5 mg L^−1^ quercetin 3,4′-glucoside.	To investigate the possible inhibitory effects of quercetin ingestion from a dietary source on platelet function (collagen-stimulated platelet aggregation and collagen-stimulated tyrosine phosphorylation).	After HQS treatment, plasma levels of quercetin did the following: - Peaked at 2.59 ± 0.42 mmol L^−1^ (*p* = 0.0001); - AUC 911.61 ± 85·17 mmol L^−1^ per min (*p* = 0.001). Plasma levels of isorhamnetin peaked after 2 h at 0.119 ± 0.02 mmol L^−1^ (HQS) and 0.0133 ± 0.04 mmol L^−1^ (LQS) (*p* = 0.0001). Plasma levels of tamarixetin peaked after 2.5 h at 0.172 ± 0.035) mmol L^−1^ (HQS) and 0.0049 ± 0.001 mmol L^−1^ (LQS) (*p* = 0.0001).	HQS treatment inhibited the following: - Collagen-stimulated platelet aggregation (time-dependent); - Collagen-stimulated tyrosine phosphorylation (*p* = 0.001). The inhibition of tyrosine phosphorylation was correlated with AUC of quercetin after HQS intake.	[40]
36 healthy human subjects (16 M; 20 F) (mean age: 31.4 ± 7.7 years) Duration: 4 weeks Randomized crossover study	Treatment period: high flavonol (HF) diet based on daily consumption of 150 g onion cake (89.7 mg quercetin) + 300 mL black tea (1.4 mg quercetin). Control period: low flavonol (LF) period based on exclusion of flavonol and flavone foods and tea.	To determine the effect of dietary intake of quercetin from onions and black tea on oxidative damage to leukocytes DNA bases.	Plasma quercetin was <LOD (66.2 nmol L^−1^) after the HF period and increased at 228.5 ± 34.7 nmol L^−1^ after HF period.	The concentrations of the products of oxidative damage to DNA bases did not differ significantly between the two dietary treatment periods for any of the products measured.	[41]
32 healthy subjects (mean age 30.4 ± 7.3 years) Duration: 4 weeks Randomized crossover study	Treatment period: high flavonol (HF) diet based on daily consumption of 150 g onion cake (89.7 mg quercetin) + 300 mL black tea (1.4 mg quercetin). Control period: low flavonol (LF) period based on exclusion of flavonol and flavone foods and tea.	To investigate the effects of a high-flavonoid (HF) diet on markers of oxidative stress (F2 -isoprostanes and malondialdehyde (MDA)-modified LDL) compared with a low-flavonoid (LF) diet.	After the HF treatment, plasma quercetin concentrations were significantly higher (221.6 ± 37.4 nmol L^−1^) than after the LF treatment (compared with less than the LOD of 66.2 nmol L^−1^).	There were no significant differences in plasma F2-isoprostane concentrations, and MDA–LDL between the HF and LF dietary treatments.	[42]
229 healthy subjects (mean age 31.05 ± 8.9 years) Duration: 3 months Randomized double-blind, placebo controlled study	Participants consumed 16.7 mg/day of sea buckthorn extract or placebo, added to 28 g of puree. The daily dose of sea buckthorn extract contained: - 5.8 ± 0.7 mg isorhamnetin 3-O-glucoside-7-O-rhamnoside; - 1.5 ± 0.9 mg quercetin 3-Orutinoside; - 1.6 ± 0.4 mg quercetin 3-O-glucoside; - 5.1 ± 0.8 mg isorhamnetin 3-O-rutinoside; - 2.4 ± 0.4 mg isorhamnetin 3-O-glucoside; - 0.3 ± 0.4 mg kaempferol 3-O-rutinoside.	To study the effect of flavonoid-rich sea buckthorn berry on circulating lipid markers associated with CVD risk (total, HDL and LDL cholesterol, triacylglycerols) and CRP.	The consumption of sea buckthorn extract significantly modified the plasma concentration in treated group: ↑ quercetin (3.0 ng mL^−1^, *p* = 0.03); ↑ isorhamnetin (3.9 ng mL^−1^, *p* < 0.01).	Sea buckthorn extract did not affect serum concentration of any CVD risk factors considered.	[43]
15 healthy volunteers (6 M; 9 F) (mean age 60.8 ± 9.3 years) Duration: 1 week Randomized, controlled, crossover study	Each subject received 5 doses of quercetin-3-O-glucoside: - 0 mg; - 50 mg; - 100 mg; - 200 mg; - 400 mg. Each compound (control or treatment) was provided once in the morning in a cup of coffee.	To determine whether endothelial function, BP and NO were affected in a dose-dependent mode of administration of quercetin-3-O-glucoside.	After the intake of increasing doses of quercetin-3-O-glucoside, was observed: ↑ quercetin dose-dependent plasma concentrations (R^2^ = 0.52, *p* < 0.001), ↑ isorhamnetin dose-dependent plasma concentrations (R^2^ = 0.12, *p* = 0.005). Baseline: - free quercetin 1.90 ± 1.1 mM; - isorhamnetin 0.99 ± 0.06 mM.	After any intervention, no improvements were observed in: - endothelial function, - BP; - NO production.	[47]
6 healthy subjects (4 M; 2 F) (mean age 34 ± 7 years) Duration: 1 day Randomized placebo-controlled crossover study	Participants were randomly treated with the following: - 150 mg Q-4-G in 5% ethanol; - 300 mg Q-4-G in 5% ethanol; - 5% (*v*/*v*) ethanol control drink.	To investigate the effect of the dietary ingestion of quercetin on platelet function (platelet aggregation and platelet collagen-stimulated tyrosine phosphorylation).	Plasma concentrations peaked 30 min after ingestion. Group 150 mg: - Quercetin 4.66 ± 0.77 μM; - Isorhamnetin 0.16 ± 0.05 μM; - Tamarixetin 0.24 ± 0.07 μM; - Total flavonoid 5.07 ± 0.90 μM. Group 300 mg: - Quercetin 9.72 ± 1.38 μM; - Isorhamnetin 0.44 ± 0.07 μM; - Tamarixetin 0.54 ± 0.09 μM (after 45 min); - Total flavonoid 10.66 ± 1.55 μM. These results indicating dose-dependent bioavailability of flavonoid.	After 30 and 120 min since intake of both doses of Q-4-G were inhibited: - platelet aggregation (*p* = 0.001); - collagen-stimulated tyrosine phosphorylation of TPP (*p* = 0.001).	[48]
12 healthy men (mean age of 43.2 ± 4.3 years) Duration: acute consumption Randomized, placebo-controlled, crossover trial	Each participant received, in random order, 4 treatments: - 300 mL water (control); - 0.67 mg/mL quercetin; - 0.67 mg/mL epicatechin; - 0.67 mg/mL EGCG.	To evaluate the effects of quercetin and epicatechin on the endothelial function (measuring endothelin-1 and NO production) and oxidative stress (measuring urinary F2-isoprostanes).	Acute treatment with quercetin and epicatechin significantly increased (*p* < 0.001) the total circulating concentration of each flavonoid (from 0.84 ± 0.39 μmol L^−1^ to 3.54 ± 1.57 μmol L^−1^ for quercetin and from and 0.70 ± 0.34 μmol L^−1^ to 3.57 ± 1.21 μmol L^−1^ for epicatechin). In urine, concentrations of total quercetin increased from 0.61 ± 0.15 to 2.51 ± 0.65 μmol mmol^−1^ creatinine and total epicatechin from 0.50 ± 0.28 to 2.62 ± 0.98 μmol mmol^−1^ creatinine (*p* < 0.001). Plasma concentrations of EGCG increased from 0.06 ± 0.01 to 0.10 ± 0.01 μmol L^−1^ (*p* < 0.05). EGCG was not detected in urine.	EGCG did not affect NO production. Quercetin and epicatechin significantly reduced plasma endothelin-1 concentration (*p* < 0.05), but only quercetin significantly decreased the urinary endothelin-1 concentration. None of the 3 treatments significantly decreased plasma or urinary F2-isoprostane concentrations.	[49]
15 healthy subjects (9 M; 6 F) (mean age 25.8 ± 5.2 years) Duration: 3 weeks Double blind, randomized, placebo-controlled trial.	Subjects received a capsule containing the following: - Placebo; - 200 mg of quercetin; - 400 mg of quercetin.	To evaluate whether the deconjugation of quercetin-3-O-glucuronide (Q3GA) may improve vasodilator effects of quercetin.	At 2 h post ingestion, plasma levels were as follows: - 200 mg quercetin group: 0.35 μM Q3GA, 0.043 μM quercetin aglycone, 0.008 μM isorhamnetin aglycone, - 400 mg quercetin group: 0.95 μM Q3GA, 0.031 μM quercetin aglycone, 0.035 μM isorhamnetin aglycone. Glucuronides of isorhamnetin were not detected.	After ingestion (2 or 5 h) of both doses, were not changes in systolic and diastolic blood pressure. A time-dependent increase in brachial artery diameter was detected after 400 mg quercetin intake, correlated with the levels of Q3GA mediated by glucuronidase activity.	[50]

CRP, C reactive protein; CVD, cardiovascular disease; BP, blood pressure; FMD, flow-mediated dilatation; FPG, fasting plasma glucose; sBP, systolic blood pressure; HF, high-flavonoid; LF, low flavonoid; LOD, Limit of Detection; EGCG, Epigallocatechin gallate; Q-4-G, quercetin-4-O-β-glucoside; TPP, total platelet proteins.

**Table 4:** Phenolic acids’ beneficial effects and bioavailability in human subjects.

Cohort and Study Details	Phenolic Acids Intake	Aim	Bioavailability Data	Outcome	Reference
1° study: 15 healthy male subjects (mean age: 26.3 ± 1.6 years) 2° study: 24 healthy male subjects (mean age: 23.8 ± 1.4 years) Duration: acute consumption Randomized controlled crossover studies	1° study: Subjects consumed 50 mL coffee containing high (310 mg) or low (89 mg) chlorogenic acid levels. Control intervention contained 0 mg chlorogenic acid. 2° study: Subjects consumed (a) 0.45 g purified 5-caffeoylquinic acid (5-CQA) + 1 g maltodextrin (MDX); (b) 0.90 g purified 5-CQA + 1 g MDX; 1 g MDX (negative control) and 0.20 g purified epicatechin + 1 g MDX (positive control). Each preparation was solubilized in 200 mL of hot water.	To evaluate the vascular function (% flow-mediated dilation, FMD) after the consumption of coffee rich in chlorogenic acid.	1° study: After 1 h of treatment was observed, a significant positive correlation between total plasma chlorogenic acids metabolites (CgAM) and % FMD: 3-caffeoylquinic acid (3CQA) 4-caffeoylquinic acid (4CQA), 5-feruloylquinic acid (5FQA) and caffeic-3′-O-sulfate (CA3S) (*p* < 0.005). After 5 h, where a second peak of % FMD was observed, the correlation was significant for ferulic-4′-O-sulfate, isoferulic-3′-O-glucuronide, caffeic-4′-O-sulfate, m-coumaric acid-3′-O-sulfate and 4-methoxycinnamic acid (*p* < 0.005). 2° study: Total plasma CgAM post intervention of 0.45 g of chlorogenic acids were lower than following intake of the 0.90 g dose, but both significantly ↑ (*p* = 0.032 and *p* = 0.006, respectively).	1° study: Both low and high chlorogenic enriched coffee improved vascular function 2° study: Only after the 0.45 g dose of purified 5-CQA, %FMD ↑ in comparison to the water control (6.02 ± 0.28 to 6.77 ± 0.42, *p* = 0.06), whereas intake of the control (5.90 ± 0.33% to 5.86 ± 0.30%) or the higher dose of 5-CQA (0.900 g) had no effect on %FMD (6.17 ± 0.31% to 6.46 ± 0.35%).	[55]
23 healthy subjects (4 M; 19 F) (mean age: 52.3 ± 10.6 years) Duration: acute consumption Randomized, double-blind, placebo-controlled, crossover trial	The treatments included: - water (used as control); - 0.40 g of chlorogenic acid (3-O-caffeoylquinic acid) solubilized in 0.20 L of water (corresponding to 2 cups of coffee).	To investigate the acute effects of chlorogenic acid on different parameters: - Nitric oxide level; - Endothelial function; - Blood pressure.	Chlorogenic acid concentration in plasma was significantly higher 150 min after the consumption of 0.40 g of CGA as compared to control group (*p* < 0.001). No significant differences were observed in term of chlorogenic acid metabolites (isoferulic acid, ferulic acid, phloretic acid, caffeic acid, and hydrocaffeic acid) between the two treatments.	The mean systolic blood pressure (−2.41 mmHg, *p* = 0.05) and diastolic blood pressure (−1.53 mmHg, *p* = 0.04) were significantly ↓ in the chlorogenic acid group compared with control group. NO levels (*p* > 0.10) and endothelial function (*p* = 0.60) were not significantly affected.	[56]
75 healthy subjects (38 M; 37 F) (mean age: 38.5 ± 9 years) Duration: 8 weeks Randomized, placebo-controlled trial	Group A: consumption 400 mL coffee/day containing high levels chlorogenic acids (HCCGA) (780 mg/400 mL); group B: (400 mL coffee/day containing medium levels chlorogenic acid (MCCGA) (420 mg/400 mL); group C: placebo. Chlorogenic acids were composed by: 5-O-caffeoylquinic; 3-O-caffeoylquinic; 4-O-caffeoylquinic; caffeic; ferulic; 3,4-di-O-caffeoyl-quinic; 3,5-di-O-caffeoylquinic; and 4,5-di-O-caffeoylquinic.	To evaluate the effects of chlorogenic acids on the following: - The antioxidant capacity of plasma; - The lipid profile in serum; - The vascular function (flow-mediated dilation-FMD, nitric oxide levels, blood pressure).	The concentration of ferulic and caffeic acid was ↑ in the groups that consumed coffee-drinking and were significantly higher 1 h after the consumption in the MCCGA group than the HCCGA group (caffeic acid: 50.5 ± 6.9 nM vs 20.3 ± 3.3 nM; ferulic acid: 201 ± 18.7 nM vs. 137 ± 6.1) (*p* < 0.001). In both groups the ferulic and caffeic acids decreased after 8 weeks.	Plasma antioxidant capacity significantly increased only in the group consuming medium levels of chlorogenic acid (6%) and group consuming high levels chlorogenic acids (5%) (*p* < 0.05). No effects on lipid profile and vascular function were measured.	[59]
16 healthy subjects (10 F, 6 M) (mean age: 59.9 ± 8.2 years) Duration: acute consumption Double-blind, randomized, placebo-controlled crossover study	Subjects received: (1) 0 g purified 5-chlorogenic acid (p5-CgA) (control group); (2) 0.45 g p5-CgA; (3) 0.90 g p5-CgA; and (4) 0.20 g purified (−)-epicatechin (positive control). Each treatment was prepared in 200 mL of warm water and consumed within 10 min. A minimum one-week washouts between visits was required.	To evaluate the acute effect of two doses of (5-CgA) (0.45 and 0.90 g) on vascular function (FMD) and blood pressure.	After 1 h and 4 h from the consumption of 0.90 g 5-CgA, total CgA metabolites, reached 1.5 μM and 1.25 μM, respectively. After 0.45 g, metabolites reached 0.75 μM and 1 μM after 1 and 4 h, respectively. In both cases, the most representative CgA metabolite was 5-caffeoylquinic acid (5-CqA).	None of the doses of 5-CgA used significantly affected FMD response.	[63]
12 healthy subjects (5 F, 7 M) (mean age: 59.4 ± 6.4 years) Duration: acute consumption Randomized, placebo-controlled, crossover trial	Subjects consumed: (1) 18 g of ground caffeinated coffee + 0.3 g CgA in 0.20 L of hot water; (2) 18 g of decaffeinated coffee + 0.287 g CgA in 0.20 L of hot water; (3) 0.20 L of hot water (control). For each group the beverages were consumed twice. The second beverage was consumed after 2 h with a 75 g glucose load.	The aim of the study was to evaluate the effect of coffee on different outcomes: (1) vascular function (FMD), (2) blood pressure, (3) glucose concentration in blood.	The mean values concentration for 5-CgA were: - caffeinated group: 1.89 ± 0.56 nM; - water group: 1.21 ± 0.22 nM; - decaffeinated group: 1.30 ± 0.29 nM. No significant difference in 5-CgA concentration between the three groups were observed.	The FMD response was significantly ↑ in the caffeinated coffee group compared to the other groups (decaffeinated coffee and water) (*p* < 0.001). No significant difference in the FMD response between decaffeinated coffee and water groups was observed. No differences were observed in term of blood glucose concentrations and blood pressure between the three groups considered.	[60]
68 healthy overweight/obese subjects (23 M; 45 F) (mean age: 38.5 ± 2 years) with sedentary lifestyle and reduced intake of fruit and vegetables Duration: 8 weeks Randomized placebo-controlled, randomized trial	Whole grain (WG) group consumed 70 g wheat/day containing 96.7 mg ferulic acid, 26.5 mg sinapic acid, 9.4 mg coumaric acid, 1.9 mg gallic acid, 1.8 mg syringic acid, 1.6 mg vanillic acid, 0.5 mg salicylic acid, 0.3 mg caffeic acid); control group (CTR) consumed 60 g refined wheat (RW) products/day containing 2.6 mg ferulic acid.	To investigate the role of whole grain (WG) consumption on plasma markers of metabolic disease and inflammation (tumor necrosis factor-α (TNF-α) interleukin-10 (IL)-10), plasminogen activator inhibitor 1.	After 8 weeks, WG consumption was associated with a 4-fold ↑ in serum dihydroferulic acid (DHFA) and a 2-fold ↑ in fecal ferulic acid (FA) compared with RW group. Similarly, after 8 weeks, urinary FA was 2-fold ↑ the baseline concentration only in WG group.	In the WG group was observed a ↓ in TNF-α after 8 weeks and ↑ IL-10 only after 4 weeks, compared with RW group (*p* = 0.04). No significant differences in plasma metabolic disease markers were observed.	[62]
47 habitual coffee drinkers at risk for type-2 diabetes (11 M; 36 F) (mean age 54.0 ± 9.0 years) Duration: 3 months Crossover clinical trial	The coffee consumption during the trial was set as follow: - First month: subjects avoided to drink coffee; - Second month: subjects consumed 4 cups (150 mL/cup) of filtered coffee/day; - Third month: participants consumed 8 cups of filtered coffee/day.	To evaluate the role of daily coffee consumption on the following: - The modulation of different biomarkers of inflammation (interleukin-18 (IL-18), 8-isoprostane, and adiponectin); - Oxidative stress and glucose; -Lipid metabolism.	Positive correlations between the ↑ in serum concentrations of coffee phenolic acid metabolites (cPAM) and changes in adiponectin concentrations were detected after 8 cups coffee/day consumption vs. baseline (0 cups coffee/day). Significant correlations were observed for isoferulic acid (*r* = 0.328, *p* = 0.025) and dihydroisoferulic acid (*r* = 0.323, *p* = 0.027). Negative or no correlation was observed for the other cPAM.	Significant differences were observed for serum concentrations of IL-18, 8-isoprostane, only after 8 cups coffee/day consumption compared with baseline (0 cups coffee/day). Serum concentrations of total cholesterol, HDL cholesterol, and apolipoprotein A-I ↑ significantly (+12%, 7%, and 4%, respectively). The ratios of LDL to HDL cholesterol and of apolipoprotein B to apolipoprotein A-I significantly ↓ (−8% and 9%, respectively) after the consumption of 8 cups coffee/day compared with 0 cups coffee/day. No variations were observed for glucose metabolism markers.	[64]
20 healthy subjects (6 M; 14 F) (mean age: 35.7 ± 9.0 years) Duration: acute consumption Randomized placebo-controlled trial	0.4 L of Arabica coffee containing 0.42 g CgAs (6 g/100 mL: provided 105 ± 4.1 mg of CgAs) were given to the intervention group The sum of the following acids contributed to the total CgAs and phenolic acids content: 5-O-caffeoylquinic; 3-O-caffeoylquinic; 4-O-caffeoylquinic; caffeic; ferulic; 3,4-di-O-caffeoylquinic; 3,5-di-O-caffeoylquinic; and 4,5-di-O-caffeoylquinic. No coffee or placebo were consumed by the control group.	The caffeic acid (CA) and ferulic acid (FA) were quantify and different methods were applied to evaluate the antioxidant capacity of plasma.	The concentrations of FA and CA, measured 1 h after the coffee consumption, were: 202.38 ± 12.87 nM and 49.76 ± 6.44 nM, respectively. Both acids (CA and FA) were not detected at baseline. In the control group, at 1 h, the same trend was observed.	The antioxidant capacity of plasma was measured with ferric reducing antioxidant power assay (FRAP) and oxygen radical absorbance capacity assay (ORAC). Compared to the baseline, the antioxidant capacity, measured with both methods, ↑ significantly (+6.67%; *p* < 0.001 for FRAP and +7.16%; *p* < 0.05 for ORAC). A correlation was observed between the ↑ of antioxidant capacity and the plasma concentration of FA and CA.	[65]
21 healthy men (mean age: 27 ± 1.3) Duration: acute consumption Randomized, controlled, double-blind, crossover human- intervention trials	1° study: participants consumed: - A blueberry drink containing: 0.766, 1.278, and 1.791 g total blueberry polyphenols (corresponding to 0.24, 0.40, and 0.56 kg fresh blueberries, respectively); - Control drink (macronutrient and micronutrient drink). 2° study: participants consumed the following: - A blueberry drink containing 0.319, 0.637, 0.766, 1.278 and 1.791 g total blueberry polyphenols (corresponding to 0.10, 0.20, 0.24, 0.40, and 0.56 kg fresh blueberries, respectively); - Control drink (macronutrient and micronutrient drink).	The endothelial function (FMD) was monitored and the time-dependent (1° study) and intake-dependent (2° study) changes were investigated.	A correlation between the FMD ↑ and the plasma concentration ↑ of different phenolic acid metabolites (ferulic acid, isoferulic acid, vanillic acid, 2-hydroxybenzoic acid, benzoic acid, and caffeic acid—sum of conjugated and nonconjugated compounds) were observed 2 and 6 h after consumption. The phenolic acid metabolites were significantly ↑ at 1–2 h after blueberry polyphenol consumption (*p* < 0.001). The plasma total concentration of metabolites was about 400 nmol/L (coinciding with the highest FMD value at 1 h). The metabolites of flavanol or anthocyanin were not detected in plasma at any time after the intake of blueberry drink.	The consumption of 0.10–0.24 kg blueberry (corresponding to 0.319, 0.639 and 0.766 g total polyphenols) positively affected vascular function.	[61]

CgA, Chlorogenic Acid; HCCGA, high levels chlorogenic acids; MCCGA, medium levels chlorogenic acid; WG, whole grain.

**Table 5:** Stilbenes’, isoflavones’, and flavanones’ beneficial effects and bioavailability in human subjects.

Cohort and Study Details	Phenols Intake	Aim	Bioavailability Data	Outcome	Reference
**Stilbenes**					
60 subjects (9 M; 51 F) (mean age: 20.52 years) Duration: 28 days Randomized, double-blind, placebo-controlled, parallel-groups study	500 mg/day of pure trans-resveratrol (also containing 10 mg of piperine/capsule) or a placebo.	To evaluate the effect of resveratrol on cognitive performance (measured as serial subtractions, rapid visual information processing, 3 Back test), mood, sleep quality and cerebral blood flow (CBF).	Resveratrol 3-O-sulfate was the predominant metabolite in all volunteers, contributing 73–77% of total metabolites, followed by resveratrol 4′ glucuronide and 3′ glucuronide. Total resveratrol metabolites increased in plasma from 3 to 13 μM 110 min after administration.	Although stilbene metabolite levels increase in plasma, supplementing with 500 mg of resveratrol for 28 days did not improve cognitive function.	[68]
22 healthy subjects (4 M; 20 F) (mean age: 20.17 years) Duration: 7 days Randomized, double-blind, placebo-controlled, crossover study	Subjects received the following treatments: - inert placebo; - 250 mg trans-resveratrol; - 500 mg trans-resveratrol;	To evaluate the effects of oral resveratrol on cognitive performance and CBF. In a separate group (*n* = 9) was investigated plasma levels of resveratrol and its conjugates after the intake of the same treatments.	Resveratrol sulfate and glucuronide were the main metabolites and reached a peak plasma concentration at 90 min after both 250 and 500 mg supplementation. As regards unmetabolized resveratrol, 90 min after both supplementations, reached low concentrations, peaking at 5.65 and 14.4 ng mL^−1^, respectively.	CBF increased in a dose-dependent fashion of resveratrol intake during task performance. No changes in cognitive function were registered.	[69]
**Isoflavones**					
10 overweight or obese men (mean age: 56.2 ± 6.18 years) Duration: acute consumption Randomized, double-blind, placebo-controlled, crossover study	Subjects consumed a high-fat, high-fructose breakfast with 4 dietary supplementations: - Placebo: fish oil placebo and isoflavone placebo; - FO: fish oil and isoflavone placebo; - ISO: fish oil placebo and isoflavones; - FO + ISO: fish oil and isoflavones. The soy isoflavone supplements provided 150 mg glycoside isoflavones (eq. to 96 mg aglycone form) in proportions of 1.05/1.0/0.29 for genistein/daidzein/glycitein. Fish oil supplement (1 g of refined fish oil concentrate) providing 0.4 g EPA and 0.2 g DHA.	To evaluate the effect of acute supplementation with fish oil (*n*-3), PUFA, soy isoflavones, and their combination on postprandial serum triglycerides (TG) and oxidative biomarkers in a proatherogenic high-fat, high-fructose meal.	At 4 h, postprandially serum concentration was as follows: - Genistein ISO: 1.027 ± 0.122 μmol L^−1^, FO + ISO: 1.185 ± 0.079 μmol L^−1^; - Daidzein ISO: 0.838 ± 0.096 μmol L^−1^, FO + ISO: 1.017 ± 0.046 μmol L^−1^.	The high-fat, high-fructose meal significantly increased serum total FA and TG without affecting oxidative stress biomarkers. Serum TG and oxidative stress biomarkers did not differ between treatments. The FO and ISO were bioavailable but did not reduce the postprandial rise in serum TG. Neither the study meal nor the FO or ISO induced significant changes in oxidative stress.	[71]
78 postmenopausal osteopenic women (mean age: 61.85 ± 1.03 years) Duration: 12 months Double-blind, parallel design, placebo-controlled trial	Participants received supplementation: 1.2 g/day calcium, 0.55 g/day magnesium, 0.025 g/day calcitriol, and a red clover extract (0.06 g/day isoflavone aglycones and probiotics) or a placebo.	To determine the beneficial effects of a bioavailable isoflavone and probiotic treatment in postmenopausal osteopenia.	After 12 month, isoflavone concentration in the treated group was 3.933 μg mL^−1^ (median), significantly higher from baseline (*p* = 0.0094) (compared with the control group, where median values were 2.323 μg mL^−1^).	Treatments with red clover extract: ↓ BMD loss, ↓ plasma concentrations of collagen type 1 crosslinked C-telopeptide (*p* < 0.05).	[72]
237 women (mean age: 53 ± 3 years) Duration: 12 months Randomized, double-blind, placebo-controlled, parallel, multicenter trial (including the Netherlands, Italy and France)	Subjects, during their habitual diet and lifestyle, consumed 110 mg/day isoflavone aglycones or control.	To evaluate whether bone metabolism and mineral density, and hormonal conditions were affected by chronic consumption of isoflavone-enriched foods.	↑ isoflavones plasma levels in treated group. Both genistein and daidzein were higher in the Netherlands (1522 ± 1136.2 nmol L^−1^ and 338.2 ± 261.3 nmol L^−1^, respectively) than in France (533.4 ± 607.4 nmol L^−1^ and 92.9 ± 145.8 nmol L^−1^, respectively) and Italy (541.5 ± 557.6 nmol L^−1^ and 133.4 ± 188.3 nmol L^−1^, respectively).	Bone mineral density or biomarkers of bone were not affected by isoflavone-enriched products chronic intake. Hormone concentrations did not differ between the two groups.	[73]
**Flavanones**					
16 men at moderate CVD risk (mean age: 60.6 ± 1.4 years) Duration: acute consumption Randomized, placebo-controlled crossover trial	Participants received 767 mL orange juice or a hesperidin supplement (both providing 320 mg hesperidin and 439 mg vitamin C) or control.	To evaluate the effects of orange juice or a hesperidin supplement on plasma concentrations of flavanone metabolites and their effects on cardiovascular risk biomarkers (blood pressure, endothelial function, central arterial stiffness, cardiac autonomic function, platelet activation, and NADPH oxidase gene expression).	After 5 h from the orange juice intake, significantly increased plasma concentrations of 8 flavanones (1.75 ± 0.35 mmol L^−1^, *p* < 0.0001), and 15 other phenolic metabolites (13.27 ± 2.22 mmol L^−1^, *p* < 0.0001) were significantly increased. In particular, 47% hesperidin-glucuronide, 15% naringenin-7-O-glucuronide, 14% a second hesperidin-glucuronide, 54% hippuric acid, 15% dihydroferulic acid, 8% dihydroferulic acid–glucuronide, 7% 4-hydroxyphenylacetic acid, and 5% vanillic acid increase were detected.	Effects on CVD risk factor were not observed.	[74]

BMD, bone mineral density; CBF, cerebral blood flow; PUFA, polyunsaturated fatty acids; EPA, eicosapentaenoic acid; DHA, docosahexaenoic acid; NADPH, nicotinamide adenine dinucleotide phosphate.

## Data Availability

No new data were created or analyzed in this study. Data sharing is not applicable to this article.
